# Editorial: Insights in: cognition 2021

**DOI:** 10.3389/fpsyg.2023.1288388

**Published:** 2023-09-27

**Authors:** Bernhard Hommel, Ulrich Hoffrage, Claudia Repetto, Yann Coello

**Affiliations:** ^1^Department of Psychology, Shandong Normal University, Jinan, China; ^2^Faculty of Business and Economics, Université de Lausanne, Lausanne, Switzerland; ^3^Psychology Department, Catholic University of the Sacred Heart, Milan, Italy; ^4^Cognitive and Affective Sciences Laboratory-SCALab, Université Lille Nord de France, Lille, France

**Keywords:** cognition, dyscalculia development, real-world research, cognitive assistance systems, consciousness, empirical aesthetics, automatized stimulus-driven attention

We are now entering the third decade of the twenty-first century, and, especially in the last years, the achievements made by scientists have been exceptional, leading to major advancements in the fast-growing field of Psychology. Frontiers has organized a series of Research Topics to highlight the latest advancements in science in order to be at the forefront of science in different fields of research. This editorial initiative of particular relevance, led by Hommel, Specialty Chief Editor of the section Cognition, and the additional editors of this year's edition, Hoffrage, Repetto, and Coello, is focused on new insights, novel developments, current challenges, latest discoveries, recent advances, and future perspectives in the field of Cognition. Also, high-quality original research manuscripts on novel concepts, problems, and approaches were welcomed.

This Research Topic has solicited a number of brief, forward-looking contributions from the editorial board members that describe the state of the art and that outline recent developments and major accomplishments that have been achieved and/or that need to occur to move the field forward. The contributors were invited to tell us: What do you think is important? Authors were encouraged to identify the greatest, scientifically most important challenges in their sub-discipline or area, and how to address those challenges. What can we learn from the past? Where to head next?

Numerous teams of authors have responded to our challenge. In the end, eight articles could be included in this compilation. Ansorge, Baier et al. have tackled the question of how our perception is affected by language. They introduce a new concept: LASA, which stands for language-induced automatized stimulus-driven attention. They make a strong case for linguistic relativity and plea for an integrated view on the relationship between language and attention. Ansorge, Pelowski et al. point out that empirical aesthetics might hold strong potential for our understanding of human consciousness. They argue that empirical aesthetics provide a much more natural, and presumably more valid, approach to human consciousness than do artificial laboratory tasks. More generally, they plea for a more visible role of art in scientific research. Yankulova et al. focus on a new experimental instrument to investigate insuppressible cognitions: the reflexive imagery task. They highlight the promises of this task and discuss key findings. They dwell on the theoretical implications of these findings and discuss future directions in the study of human consciousness. Daprati and Nico make a bold case to emphasize the importance of studying interindividual differences. They discuss ritualistic behavior and body-size illusions as examples of how behavior in healthy individuals may be taken to fall onto the same dimension on which the more pathological extremes of such behavior can be described. The authors argue that moral consideration of interindividual differences can inform research on vulnerability factors and neuropsychiatric disorders.

Pitt and Casasanto argue that research on spatial stereotypes and concepts can provide important information for the design of everyday things. They point out that previous research on user interfaces has relied too much on verbal descriptions and verbalizable concepts, and they plea for a stronger consideration of spatial metaphors. They propose a principle for how the spatial metaphors that people use to organize their non-spatial concepts may be predicted. Strenge and Schack discuss how the design of cognitive assistance systems can benefit from cognitive sciences. They point out four big questions that need to be tackled to make assistance systems really useful: what action should be executed, when the action should be executed, whether assistance in executing the action is needed, and how execution of the action should be supported. They discuss several ways of how these questions can be successfully tackled. Carr et al. emphasize the usefulness of real-world tasks for understanding human cognition. They discuss the pros and cons for laboratory research and for real-world research, and they conclude that the integration of both kinds of investigations will be necessary to achieve scientifically and societally important goals. Finally, Dowker reviews research on typically developing children and adults and individuals with developmental and acquired dyscalculia and concludes that arithmetical ability is not a unitary psychological function but, rather, made up of various kinds of components, including symbolic and non-symbolic quantity representation and processing, and also general abilities like attention, cognitive control, and working memory. The author emphasizes the importance of further research studying the interactions between domain-specific and domain-general cognitive functions.

Taken altogether, the goal of this special edition Research Topic is to shed light on the progress made in the past decade in the field of biological or artificial Cognition, highlight future challenges, and provide a thorough overview of the state of the art in this area of research. We hope that this Research Topic will inspire, inform, and provide direction and guidance to researchers in the field.

## Author contributions

BH: Conceptualization, Writing—original draft, Writing—review and editing. UH: Writing—review and editing. CR: Writing—review and editing. YC: Writing—review and editing.

